# Radiation Induced Skin Fibrosis (RISF): Opportunity for Angiotensin II-Dependent Intervention

**DOI:** 10.3390/ijms25158261

**Published:** 2024-07-29

**Authors:** Patricia F. Boothe, Vidya P. Kumar, Yali Kong, Kan Wang, Howard Levinson, David Mu, Milton L. Brown

**Affiliations:** 1 Department of Internal Medicine, Macon & Joan Brock Virginia Health Sciences at Old Dominion University, Norfolk, VA 23507, USA; 2 Armed Forces Radiobiology Research Institute, The Uniformed Services University of the Health Sciences, Bethesda, MD 20889, USA; 3 Department of Biomedical and Translational Sciences, Macon & Joan Brock Virginia Health Sciences at Old Dominion University, Norfolk, VA 23507, USA; kongy@odu.edu (Y.K.); mud@odu.edu (D.M.); 4 The Center for Plastic Surgery at Sentara, 301 Riverview Ave. #400, Norfolk, VA 23510, USA; hxlevins@sentara.com; 5 Leroy T. Canoles Jr. Cancer Research Center, Macon & Joan Brock Virginia Health Sciences at Old Dominion University, Norfolk, VA 23507, USA

**Keywords:** radiation, skin fibrosis, reactive oxygen species (ROS), angiotensin II (Ang II), AT1R, telmisartan

## Abstract

Medical procedures, such as radiation therapy, are a vital element in treating many cancers, significantly contributing to improved survival rates. However, a common long-term complication of such exposure is radiation-induced skin fibrosis (RISF), a complex condition that poses substantial physical and psychological challenges. Notably, about 50% of patients undergoing radiation therapy may achieve long-term remission, resulting in a significant number of survivors managing the aftereffects of their treatment. This article delves into the intricate relationship between RISF, reactive oxygen species (ROS), and angiotensin II (Ang II) signaling. It proposes the underlying mechanisms and examines potential treatments for mitigating skin fibrosis. The primary goal is to offer essential insights in order to better care for and improve the quality of life of cancer survivors who face the risk of developing RISF.

## 1. Introduction

It is a daunting reality that as of January 2022, the United States is home to 18.1 million cancer survivors, with a projected increase to 26 million by 2040. Radiation therapy [1,2,3] has become a cornerstone in the treatment of various malignancies, and a significant portion of cancer patients will undergo radiation therapy either as a standalone treatment or in conjunction with chemotherapy [4,5]. Among these patients, approximately half will achieve long-term cancer remission, but around 5–10% will develop serious late complications as a result of radiation therapy [4,6]. Due to the treatment application, a high volume of these cases will be skin related.

The skin, being the body’s largest organ, possesses a high rate of cell turnover and proliferation, rendering it particularly vulnerable to radiation-induced injury [7,8]. Its multifunctional role in protecting against external threats and maintaining homeostasis underscores its significance. It regulates body temperature, contributing to immunity, facilitating excretion, synthesizing vitamin D, providing sensory perception, preventing water loss, and serving aesthetic and social functions [9]. It is also the first organ exposed to radiation in treatment. The skin comprises an epidermal layer, a dermal layer, and a hypodermis, each with distinct functions. The epidermal layer consists of several cell layers, such as the stratum corneum in the topmost layer of the epidermis. The stratum corneum is formed by a specialized form of cell death, i.e., corneoptosis [10]. The stratum granulosum is three to five cell-layers thick and contains the tight junctions, stratum spinosum, and stratum basalis. The stratum basalis (also known as the basal layer or stratum germinativum) of the epidermis contains melanocytes, undifferentiated keratinocytes, melanocytes, Merkel cells, and stem cells. These stem cells are crucial for the continuous regeneration and repair of the epidermis. As a whole, all these sublayers of cells are in a state of rapid division. Due to their high rate of cell division, these cells are particularly susceptible to radiation therapy. The major effect of ionizing radiation on tissues is the direct killing of cells by damaging the DNA, resulting in cell death. Radiation-induced ionization can act indirectly, producing free radicals from the water content of a cell. Thus, it is not surprising that rapidly dividing cancer cells are vulnerable to ionizing radiation. However, it has been shown that cancer stem-like cells within solid tumors are radioresistant [11,12]. It is thought-provoking to hypothesize that radiation therapy, while extensively damaging cells in the previously discussed layers, might leave a small, vital population of stem cells unaffected [13,14,15,16]. This speculation arises from the known ability of the skin to self-renew, a process largely dependent on these stem cells [17]. In intact skin, a dynamic equilibrium is maintained by the regenerative capacity of resident stem cells, which proliferate to produce new cells, compensating for those shed through natural senescence or lost to injury [18]. This regenerative process preserves the structural and functional integrity of the skin. However, exposure to ionizing radiation can perturb this equilibrium, despite the inherent resistance of stem cells to radiation. Ionizing radiation impairs skin architecture, alters the expression and function of vital proteins, and disrupts the signaling milieu. This disruption has downstream effects on the crucial regulatory mechanisms of stem cell activity, potentially leading to skin fibrosis. Consequently, the skin’s ability to regenerate is compromised, underscoring the pivotal role stem cells play in maintaining skin health and promoting its recovery post-injury.

Directly beneath the epidermis lies the dermis, which has two main layers: the papillary and the reticular layers. The basement membrane separates the epidermis from the dermis [9,12,19]. The dermis houses a variety of cells, including fibroblasts, endothelial cells, pericytes, and smooth muscles. Endothelial cells line blood vessels and regulate blood flow, while pericytes play a crucial role in blood vessel stability, blood flow, and potentially tissue regeneration through paracrine signaling [17,20,21,22,23]. Additionally, the dermal layer hosts various structures such as arrector pili muscles, sweat glands, excretory ducts, sensory receptors, sebaceous glands, and dermal papilla [9].

The hypodermis, located below the dermis and mainly composed of adipose tissue, will not be discussed further in this context [24,25,26]. Radiation-induced fibrosis in skin and muscle is marked by a pathological wound healing process, leading to excessive deposition of the extracellular matrix (ECM). As a result, patients often experience pain, stiffness, and a significant reduction in functional ability [27].

The renin–angiotensin–aldosterone system (RAAS) regulates blood pressure homeostasis and vascular injury and repair responses [28]. Supplementary Table S1 provides more info on RAAS-related factors. There are two types of angiotensin—angiotensin I (Ang I), which is 10 amino acids long, and angiotensin II (Ang II), with 8 amino acids. In view of these three considerations, namely (i) that the RAAS plays a significant role in fibrosis [29], (ii) that Ang II is considered the most important molecule in the RAAS signaling pathway [30], and (iii) that the RAAS signaling pathway has been proven to be amenable to drug intervention [10], we devote a significant portion of this manuscript to Ang II, making a case to support the notion that drugs targeting Ang II signaling would be promising contenders for managing RISF. Indeed, the identification of Ang II receptors in the skin and other organs [31,32,33,34,35,36,37] opens the door to potential new therapies for conditions like RISF. The presence of key components of the RAAS, including Ang II, angiotensinogen, renin, and angiotensin-converting enzymes (ACE), has been identified in human skin [38]. Specifically, keratinocytes, which are the predominant cells in the epidermis, have been found to be rich in angiotensin II type 1 receptors (AT1R) as well as MAS receptors, which are part of the G protein-coupled receptor family [39]. Additionally, research has demonstrated that human keratinocytes cultured in vitro are capable of expressing Ang II. Beyond the epidermis, AT1R has also been detected in the sweat glands, and their expression extends to the dermal layer and the blood vessels within the skin [39,40].

Radiation therapy can be likened to a two-edged sword. On one side, it serves as a powerful tool in the battle against cancer. On the other, it can also inflict severe and sometimes irreversible damage to various tissues and organs. Radiation-induced skin damage can manifest along a spectrum from acute to chronic, with fibrosis occurring in the chronic phase. Acute symptoms often become visible within hours to a month after radiation therapy and can be graded for severity according to several different systems ranging from Grade 1, which is associated with erythema, dry desquamation, decreased sweating, and pigment changes, to Grade 4, characterized by ulceration, necrosis, bleeding, and non-healing skin fibrosis [4,41,42,43].

## 2. Ionizing Radiation Induces Oxidative Stress

Exposure to ionizing radiation generates reactive oxygen species (ROS). ROS represent a subset of oxygen-containing free radicals, including superoxide, hydrogen peroxide, and the hydroxyl radical, with the latter being responsible for the majority of total damage in radiation exposure (60–70%). Free radicals are short-lived but highly unstable compounds characterized by unpaired electrons [44,45].

ROS play essential roles in the body, serving as cellular messengers in redox signaling and participating in the immune system’s defense against invading pathogens [46], differentiation, proliferation, and apoptosis [47]. However, a significant problem arises when there is excessive production and accumulation of ROS, which occurs in radiation exposure. This excess of free radicals, particularly ROS, can overwhelm the body’s endogenous systems responsible for regulating their effects (superoxide dismutase, catalase, and the glutathione peroxidase system) [45,48,49].

**Figure 1:**
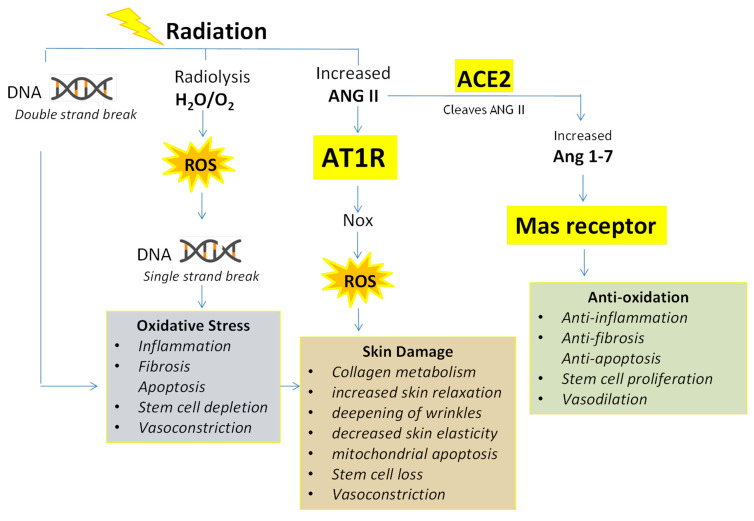
**Effects of IR on RAAS and ROS in the Skin:** Exposure to IR elicits a spectrum of genetic and phenotypic modifications within the skin. One notable effect is the augmented production of Ang II, which, upon binding to AT1R, stimulates the activity of NADPH oxidase (NOX), leading to increased levels of ROS. These elevated ROS levels are associated with various phenotypic alterations in skin tissue as detailed in the above figure. Concurrently, the rise in Ang II levels also promotes the production of Ang 1–7, which exerts its effects through MAS receptors. Ang 1–7 counterbalances the oxidative stress by offering anti-oxidative (anti-inflammatory, anti-fibrotic, anti-apoptotic), stem cell proliferative, and vasodilatory actions. Additionally, radiolysis contributes to ROS generation, further instigating DNA damage and exacerbating oxidative stress.

ROS are generated by the radiolysis of water in the nucleus and cytoplasm (Figure 1). Following ionizing radiation (IR), ROS—including the superoxide anion (O_2_^−^), hydroxy (OH^−^), and peroxide (O_2_^−2^) free radicals—are generated within the nucleus [50] and cytoplasm [51] of epithelial cells. These highly reactive entities are directly toxic by binding to DNA, lipids, and proteins, resulting in the induction of genetic instability, apoptosis, and catastrophic damage to nearby normal tissues (Figure 1) [52]. Exposure to ^137^Cs γ rays for less than a microsecond generates approximately 60 ROS per nanogram of tissue [53]. Cells irradiated with 6 GY IR demonstrated an initial rapid increase in ROS, followed by a gradual decrease in the amount of ROS over 6 h which can persist until a return to pre-IR basal levels within 2 weeks [50]. This provides the parameters for the critical window where inhibition to baseline levels can lead to genotoxicity/cytotoxicity following lethal IR.

An additional mechanism of IR-induced generation of ROS is via the activation of mitochondria [54] and cytosolic enzymes to produce free radicals. Thus, ROS generated by IR is responsible for the genotoxic and cytotoxic consequences. ROS is responsible for the hyper-inflammatory consequences, reduction of skin stem/progenitor cell survival [55], and severely impaired skin regeneration [56].

In the nucleus, IR induces damage through two mechanisms: direct damage to DNA and the creation of ROS [57,58]. The direct damage to DNA causes primarily singe-stranded breaks (SSBs) [59]. The latter mechanism is an indirect one that produces disastrous effects on DNA. As cells and tissues contain approximately 80% or more water, ionizing radiation energy results in the radiolysis of water molecules, including those surrounding DNA and within organelles like the mitochondria. This process releases a variety of products including hydroxyl radicals, hydrogen atoms, and hydrated electrons [60], of which hydroxyl radicle is the most troublesome. ROS are significant contributors to oxidative stress. They diffuse around the ionizing event in a nanometer proximity range, causing damage to the surrounding proteins, lipids, and DNA. They lead to cross-linking, damage to DNA bases, cluster DNA lesions, SSBs, and double-stranded breaks (DSBs), the latter two being determinants of cell viability and survival, since they are challenging for the repair mechanism [61]. Additionally, ROS can affect SSBs in a manner that may lead to their conversion into DSBs [53,62]. Reactive nitrogen species are also important mediators in radiation-induced injury [63,64].

Cellular contents are released as damage-associated molecular patterns (DAMPs), such as uric acid, heat-shock proteins, and HMGB1 (high mobility group box1), initiating a cascade of proinflammatory cytokines and marking the onset of the acute inflammatory process [49,65]. DAMPs are identified by pattern recognition receptors (PRRs), which are part of the toll-like receptor family. Macrophages play a central role in detecting, responding to, and orchestrating the response to these signals. They achieve this through interactions with cytokines and chemokines, which guide the immune response [66,67,68,69,70,71,72].

Additionally, research has demonstrated that ROS generated through radiation exposure can lead to the enhanced expression of various enzymes, notably cyclooxygenases (COXs), NADPH oxidase, lipoxygenases (LOXs), and nitric oxide synthase (NOS) [73]. Inhibitors of COX-2, such as celecoxib, have demonstrated a reduction in dermal inflammation and radiation-induced skin changes [74]. There is a growing body of evidence indicating the crucial role of oxidative stress in the development of late complications following radiation therapy [75,76,77,78,79,80]. While high-energy radiation can impact any part of the body, the face, neck, trunk, and extremities are more commonly affected, with approximately 90% of patients developing skin injuries after receiving radiation therapy [81,82].

There are various factors that contribute to the risk of developing severe skin injuries and, later in the timeline, RISF. The primary factor is related to the various aspects of the treatment itself: the cumulative radiation dose, dose per fraction, treated area size, and treatment duration. Notably, there is a direct correlation between the severity of radiation-induced fibrosis (RIF) and higher radiation doses, as well as the use of hypo-fractionation, larger treatment fields, and longer treatment durations [83,84,85,86,87]. RIF may occur after skin is exposed to an irradiation dose of 50 gray [88]. Other treatment-related factors that may contribute to RIF include concurrent chemotherapy use and the incorporation of surgical interventions before or after radiotherapy [89,90,91,92,93,94]. Additionally, patient-related factors, such as preexisting connective tissue diseases, can also influence the development of RIF, with patients having conditions like systemic scleroderma, systemic lupus erythematosus (SLE), or Marfan syndrome being more susceptible to severe RISF [95,96]. It should be noted that the clinical presentation of RIF varies depending on the affected tissue or organ.

It is important to note that the pathogenesis of a disease such as systemic sclerosis is multifaceted and not entirely understood and is a chronic autoimmune disease that causes extensive fibrosis, affecting the skin, internal organs, and musculature. There is increasing evidence that ROS play a critical role, particularly in the dermal fibroblasts, in the chronic inflammatory process. The dermal fibroblasts are responsible for the overproduction of extracellular matrix components, including collagen, leading to the thickening and hardening of the skin and connective tissues. This persistent oxidative stress contributes to the fibrotic process, exacerbating tissue damage and fibrosis [97,98,99].

In addition to ROS, a cohort of signaling molecules has been pinpointed as critical to the fibrotic process. These include transforming growth factor beta 1 (TGF-β1), insulin-like growth factor 1 (IGF1), chemokines CXC, tumor necrosis factor alpha (TNF-α), and, importantly, Ang II [100,101,102,103,104,105,106]. Their roles span from mediating inflammatory responses to driving tissue remodeling and fibrotic changes, thereby becoming prime targets for therapeutic strategies. Understanding the sustained impact of these molecules in chronic inflammation and their contribution to the progression of fibrosis is essential.

## 3. Clinical Manifestations of RISF

Acute radiation-induced skin changes encompass a spectrum of clinical manifestations, ranging from erythema, scaling, ulcers, pain, skin induration, dermal thickening, reduced tissue elasticity, skin and fat atrophy, hair loss, muscle shortening, pain, and contracture of the affected tissues. These manifestations collectively result in limited mobility and delayed wound healing and may progress to conditions such as radionecrosis with ulceration, lymphedema, fistula formation, hollow organ stenosis, and persistent pain [107,108,109].

Higher doses of ionizing radiation exceeding 45 GY are associated with increased severity of dermatological changes. Nonetheless, exposure to lower doses, such as 2 GY, can also induce skin changes, including transient erythema manifesting within hours after exposure [4,110]. Several grading systems exist to assess symptom severity, with the National Cancer Institute Common Terminology Criteria for Adverse Events (CTCAE) being more commonly used (Grade 0: No symptoms; Grade 1: Faint erythema, dry desquamation; Grade 2: Moderate to brisk erythema, patchy, moist desquamation within skin folds and creases, moderate edema; Grade 3: Moist desquamation (not localized to skin folds/creases), skin becomes abrased and bleeds from minor trauma; Grade 4: Ulceration/necrosis (may involve full thickness of the dermis), spontaneous bleeding, life-threatening consequences). A notable study involving 9941 breast cancer patients, conducted between 2012 and 2020, examined how patients undergoing radiation therapy reported symptoms through a questionnaire [111]. These patient-reported symptoms were then compared with clinician observations. All participants were female. The study highlighted several key insights, and patients were generally able to identify symptoms consistent with physician-established criteria. However, the study also pinpointed areas for improvement, including unreported symptoms and discrepancies in recognizing symptoms across different skin types, specifically in more highly pigmented and younger skin types [111]. Knowing where the need for improvement lies is important for future growth and improvement in patient care.

## 4. Angiotensin II and Its Connection to Oxidative Stress

The RAAS is a critical regulatory system for controlling blood pressure and fluid balance, primarily involving the kidneys, heart, and blood vessels. Historically, Ang II, an octapeptide hormone, was considered the central component of this system, acting predominantly as a vasoconstrictor through AT1R. This hormone plays a vital role in cardiac, endothelial, and renal functions. The traditional understanding of RAAS began with the conversion of angiotensinogen to angiotensin I and subsequently to Ang II. Ang II’s actions were initially thought to be mediated mainly through AT1R, which leads to vasoconstriction, and, to a lesser extent, through angiotensin II receptor 2 (AT2R), which is associated with vasodilatory effects. However, the scope of the RAAS has significantly expanded over the last twenty years with the discovery of additional angiotensin peptides, such as angiotensin 1–7 (Ang 1–7) and angiotensin 1–9 (Ang 1–9), and their respective receptors, notably the Mas receptor (Mas R). These newer components have been found to have distinct and sometimes opposing effects compared to Ang II [112].

Ang 1–7, for instance, is now recognized for its counter-regulatory actions against pro-fibrotic Ang II. Ang 1–7 is produced by angiotensin-converting enzyme II (ACE2)-directed cleavage of Ang II and exhibits vasodilatory, anti-inflammatory, and anti-fibrotic properties primarily mediated through the Mas receptor [113,114,115]. Thus, it is not surprising that an upregulation of ACE2 activity would be accompanied by an increase in Ang 1–7. It was shown in animal studies that when ACE2 was chronically inhibited, it impacted cardiac remodeling negatively and was associated with elevated levels of Ang II in the heart, which is likely due its ability to increase Ang 1–7 levels [116,117]. Moreover, ACE inhibitors and AT1R blockers have been shown in animal models to attenuate pulmonary fibrosis [112,117,118]. Radiation exposure was associated with increased ACE activity and, as previously expressed, the generation of ROS. Using an ACE inhibitor, Lisinopril, in a partial irradiation model in rats, researchers were able to show that the drug improved survival, ameliorating the effects of radiation-induced pneumonitis [119]. In another study, Captopril (ACE inhibitor) treatment was associated with a reduction in the incidence of squamous cell carcinomas of the skin and subcutaneous sarcomas, a relative consequence of receiving high doses of radiation exposure [120]. Blocking the angiotensin II receptor following total body radiation in a rat radiation nephropathy model helped to mitigate and treat radiation-induced chronic renal failure [121]. The observation that radiation generates ROS is not surprising, but it is intriguing that an ACE inhibitor may ameliorate the effects, since the generation of ROS is pivotal for the progression of fibrosis.

Another critical pathway, resulting in the excessive generation of ROS following IR, is the production of elevated Ang II (Figure 1). Studies indicate that irradiation upregulates Ang II expression in a dose-dependent manner [122]. Ang II is the key product of the RAAS that regulates intracellular and extracellular ROS and maintains blood pressure control. Unfortunately, high levels of Ang II cause multiple pro-inflammatory, pro-fibrotic, hyper-oxidative tissue-damaging effects and vasoconstriction [123]. Ang II binds to AT1R expressed on endothelial, epithelial, and fibroblast cells throughout the skin [39,40] and, as such, mediates the geno/cytotoxic features described above (see Figure 1).

Since ROS are a key risk factor contributing to RISF, we discuss the interconnections between the RAAS components and ROS in human skin cells and potential consequences of such connections. Human skin cells synthesize Ang II [38]. Both AT1 and AT2 receptors have been identified in the epidermis and in the walls of dermal blood vessels. This expression pattern is mirrored by angiotensinogen, renin, and ACE. At the mRNA level, all these components have been detected in cultured primary keratinocytes, melanocytes, dermal fibroblasts, and dermal microvascular endothelial cells, with the exception of AT2 receptors in melanocytes. The capacity of cutaneous cells to produce Ang II was confirmed by its presence in cultured keratinocytes. Moreover, in keratinocyte monolayers subjected to artificial wounds, there was a rapid increase in ACE-mRNA expression, and elevated ACE expression persisted in human cutaneous scars even three months after injury [38]. These findings indicate that the complete RAAS is present in human skin and contributes to both normal cutaneous balance and the healing of cutaneous wounds.

It has been evident that Ang II, acting through the AT1R, promotes angiogenesis, inflammation, and the migration of fibroblasts, keratinocytes, and melanocytes. As a result, it exacerbates fibrosis and contributes to scar formation in the skin [124].

**Figure 2:**
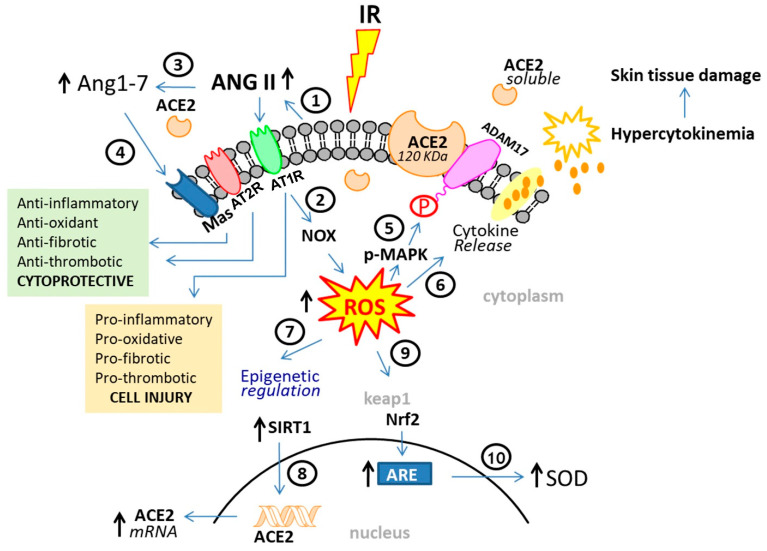
**Mechanism of Radiation-Induced Skin Damage via the Angiotensin II Pathway:** Each of the ten individual mechanistic consequences of IR on the Ang II signaling pathway is as discussed in the text under “Angiotensin II and Its Connection to Oxidative Stress”.

Exposure to IR results in a dose-dependent release of pro-inflammatory, pro-oxidative and pro-fibrotic Ang II (Figure 2, #1) [122,125,126,127]. Elevation of extracellular Ang II concentrations post IR results in the stimulation of AT1R and release of ROS [128,129] via activation of NADPH oxidase (NOX) (Figure 2, #2). During normal homeostatic conditions, the cytoprotective, anti-inflammatory, anti-fibrotic, ACE2 enzyme acts as a counterbalance to ACE by cleaving Ang II into Ang peptide 1–7 (Figure 2, #3), which binds to the anti-inflammatory, anti-oxidative, anti-fibrotic MAS receptor, reversing the harmful effects of Ang II (Figure 2, #4). Thus, ACE2 serves as the critical counter-regulatory control to prevent the tissue-damaging consequences of increased Ang II.

An interesting additional consequence of Ang II-mediated ROS activation is the finding that ROS upregulates the AT1R, adding to the tissue injury generated by excessive Ang II. The cleavage of ACE2 is mediated via ROS-dependent activation of MAP kinase which in turn phosphorylates and activates ADAM17 (Figure 2, #5), contributing to a build-up of pro-fibrotic Ang II. Elevated ROS serves as a trigger for the release of inflammatory cytokines resulting in hypercytokinemia and skin tissue damage (Figure 2, #6). Skin tissue damage is widely accepted as a direct result of inappropriate and persistent inflammatory response [130]. A key mediator in this mechanism involves a surge in ROS levels, resulting in the release of pro-inflammatory, tissue destructive cytokines (Figure 2, #6) [131,132,133,134].

Surges in cytoplasmic ROS levels also increases the epigenetic regulation of ACE2 expression (Figure 1, #7) by activation of SIRT1 via AMPK. Activated SIRT1 crosses the nuclear membrane, binds to the ACE2 promoter region, and facilitates increased ACE2 mRNA expression (Figure 1, #8). Sirt1 activation is a central mechanism of the drugs that up-regulate ACE2 expression [135,136,137,138]. Induction of ROS results in the nuclear translocation of Nrf2 [139]. Nrf2 crosses the nuclear membrane to bind with the anti-oxidant response element (ARE) (Figure 1, #9), resulting in modulated (decrease or increase) transcription of cellular detoxifying genes and anti-oxidants like superoxide dismutase (SOD) (Figure 1, #10) [140].

## 5. Angiotensin II Influences Skin Cell Behavior

Ang II has been shown to stimulate the synthesis of collagen, which is observed alongside elevated levels of TGF-β, TIMP-1, and types I and III procollagens. This increase in activity is mitigated by the administration of losartan, a blocker of AT1R [141]. Ang II plays a significant role in vascular fibrosis. Its production, triggered by the renin–angiotensin system, is pivotal in the pathophysiology of both hypertension and myocardial ischemia, in which chronic inflammation is a central theme. These disorders are associated with cardiac remodeling, which includes the hypertrophy of cardiac muscle and the proliferation of fibroblasts [142,143].

Fibroblasts and keratinocytes are essential for the wound healing process. Ang II induces keratinocytes and fibroblast migration, thus playing a critical role in skin wound healing [144]. It was shown that the targeted inhibition of AT1R through oral administration in a rat model resulted in a significant decrease of keratinocyte-driven re-epithelialization and angiogenesis, which are critical processes in cutaneous wound healing [145]. Understanding the role of angiotensin II within this context is vital for developing targeted therapies for fibrosis, particularly following radiation exposure. Our current knowledge on the subject is limited, and much more work is needed to elucidate the mechanisms involved in this intricate process. It should be noted that a significant limitation to interpreting many studies is correlated with the short half-life of Ang II, lasting only around 30 s in vascular vessels and approximately 15 to 30 min in tissues [146,147]. Additionally, its swift conversion to Ang III and Ang 1–7 in culture further complicates the process of accurately interpreting experimental outcomes [148,149].

## 6. Evidence Supporting the Renin–Angiotensin–Aldosterone System as an Entry Point to Mitigate RISF

Telmisartan, an AT1R antagonist and a partial agonist of PPARγ, has demonstrated its potential in ameliorating fibrosis in various organ systems. Previous studies have highlighted its effectiveness in reducing cardiac fibrosis through multiple pathways, including the TGF-β1/SMAD signaling pathway and the PPAR delta/STAT3 pathway, particularly in hyperglycemia-induced cardiac fibrosis [150,151,152,153,154] Moreover, telmisartan has shown promise in addressing fibrosis by inhibiting the expression of key factors such as α-smooth muscle actin, collagen 1a1, and TGF-β1 [155]. It has also been investigated for its ability to mitigate liver fibrosis associated with cirrhosis-associated portal hypertension, where it influences factors like Kruppel-like factor-4, endothelial nitric oxide synthase (eNOS), and inflammatory responses [156,157,158,159,160,161,162,163,164,165]. Despite these findings in other organ systems, there remains a need for further research to determine the potential role of telmisartan in radiation-induced fibrosis of the skin, given its integral role in attenuating fibrosis in multiple systems.

Post IR exposure, several molecular pathways are activated, resulting in increased production of ROS (Figure 1). A notable pathway involves the radiation-induced activation of the RAAS, specifically increased tissue concentration of Ang II, which, upon binding to its AT1R, initiates a cascade of intracellular events including the transcriptional and translational upregulation of the NADPH oxidase family of enzymes, designated NOX1 through NOX5 and DUOX1/2. The upregulated NOX enzymes contribute to a significant increase in ROS levels within irradiated tissues, which are instrumental in mediating some of the deleterious effects of ionizing radiation, including oxidative stress and damage to critical biomolecules.

In addition to this pathway, ionizing radiation also directly damages DNA molecules by causing single-stranded breaks in the DNA strands [166] (Figure 1). Moreover, an indirect pathway of damage involves the radiolysis of water molecules—a prevalent component of biological tissues. The radiolysis of water generates hydroxyl radicals among other reactive species, further augmenting the intracellular ROS pool [167]. Both the direct and indirect pathways of DNA and tissue damage are crucial in understanding the multifaceted nature of radiation-induced cellular injury. Collectively, the enhanced production of ROS through these mechanisms can lead to oxidative modifications of nucleic acids, proteins, lipids, and other cellular constituents [168]. These events are associated with a massive increase in cytokines that increase in quantity and duration. Together, these pathways contribute to the pathophysiology of radiation-induced tissue damage and the development of fibrosis [169].

A therapeutic compound combining AT1R antagonism with free radical scavenging capabilities offers a multifaceted approach to combat radiation-induced skin fibrosis. The mechanism above highlights what happens upon exposure to radiation. By targeting AT1R early, this drug would directly counteract the Ang II-associated upregulation of NADPH oxidase and the subsequent rise in ROS, thereby eliminating or ameliorating the fibrogenic effects of Ang II. This includes the critical task of inhibiting the overproduction of TGF-β, which, as mentioned earlier, is a central cytokine in promoting fibroblast activity for the synthesis of collagen and other extracellular matrix components. By hindering these processes, the agent is expected to curtail collagen accumulation, effectively slowing down or reversing fibrosis progression. Furthermore, the AT1R inhibition characteristic of this medication would likely manifest anti-inflammatory properties, diminishing the inflammatory milieu that results from the overexpression and prolonged activity of numerous cytokines, which are key players in fibrogenic pathways, and their modulation by this drug could significantly reduce the intensity and extent of fibrotic alterations in the skin post radiation exposure.

The drug’s free radical scavenging attribute is particularly crucial in addressing both direct and indirect oxidative stress induced by ionizing radiation. This stress is a primary factor in cellular damage and death, which can precipitate fibrotic remodeling of the skin. By neutralizing free radicals originating from principal sources, the drug would safeguard cellular structures against oxidative harm. This protective action would also mitigate the inflammatory response typically associated with radiation exposure.

**Figure 3:**
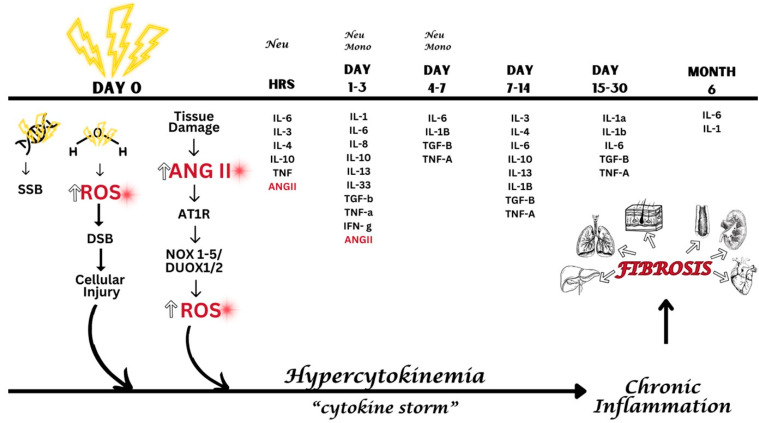
**Proposed Rearticulation of the Mechanism for Radiation-Induced Fibrosis**: Initial radiation exposure initiates at least three distinct pathways. First, radiation directly compromises DNA integrity, manifesting as single-stranded breaks (SSBs). Second, the radiolytic decomposition of water generates ROS, which further exacerbates DNA damage through the formation of double-stranded breaks (DSBs), thereby disrupting cellular functionality. Third, radiation injures tissues, prompting an upsurge in the expression of ANG II, AT1R, nicotinamide adenine dinucleotide phosphate (NADPH) oxidase (NOX 1–5), and dual oxidases 1/2 (DUOX1/2), leading to an augmented ROS production. These mechanistic pathways collectively escalate cytokine levels, a condition termed hypercytokinemia. Without resolution, this state progresses to chronic inflammation and, ultimately, to fibrosis. Neu—neutrophils; Mono—monocytes.

By combining these dual actions—AT1R blockade and free radical neutralization—the medication would not only address the onset of fibrosis but also aid in the resolution of existing fibrotic tissues. This synergistic approach in a single pharmaceutical agent marks a potentially substantial progression in treating and managing radiation-induced skin fibrosis, offering a more comprehensive and effective therapeutic strategy (Figure 3).

## 7. Conclusions

Finding new therapeutics for RISF holds significant clinical importance for enhancing the quality of cancer care. Radiation therapy, a cornerstone in oncologic treatments, often results in skin fibrosis, which manifests as hardened, less pliable tissue, leading to discomfort, disfigurement, and impaired mobility in the affected area. Clearly, there is an urgent need for new therapeutics for this debilitating disease. In this review, we present evidence that dysregulation of RAAS and ROS contributes to RISF. Consequently, we propose that dual-action small molecules antagonizing RAAS and ROS would be strong and novel candidates for drug development to manage RISF. We invite others to join us in the campaign to explore and create new strategies to combat RISF.

## Data Availability

The datasets and materials used and/or analyzed during the current study are available from the corresponding author upon reasonable request.

## Supplementary Materials

The following supporting information can be downloaded at: https://www.mdpi.com/article/10.3390/ijms25158261/s1. References [170,171,172,173,174,175,176,177,178,179,180,181,182,183,184] are cited in the Supplementary Materials.
