# Supplementary material for: Radiation Induced Skin Fibrosis (RISF): Opportunity for Angiotensin II-Dependent Intervention
Source: Int J Mol Sci. 2024 Jul 29;25(15):8261. doi: 10.3390/ijms25158261 (PMC11312688; doi:10.3390/ijms25158261)
Supplement: Supplementary file 1 [file ijms-25-08261-s001.zip › ijms-3115992-supplementary.pdf]

# **Table S1**: Molecular Properties of Key Molecules of the RAAS

| | Renin | Angiotensi-nogen | Ang I | Ang II | Ang III | Ang IV | Ang 1-7 | Ang 1-9 | Aldosterone |
|---|---|---|---|---|---|---|---|---|---|
| **Receptor** | (Pro)renin receptor, also known as ATP6AP2 | None | This peptide does not have any known biological activity [170]. | Ang II AT1R and AT2R Ang II> Ang III> Ang IV [170] | Ang II AT1R and AT2R | AT4 receptor [171, 172] | Mas receptor | AT2R | mineralocorticoid receptor (MR) |
| **Cell*** | Renin cleaves angiotensinogen to form Ang I; smooth muscle cells, alveolar cells type 2, connective tissue cells # | cleaved by renin to form Ang I; secreted to blood # | Cleaved by ACE to form Ang II. Vascular smooth muscle cells; Endothelial cells | Vascular smooth muscle cells; Endothelial cells | Vascular smooth muscle cells; CNS, Endothelial cells | porcine pulmonary endothelial cells, rabbit cardiac fibroblast [171] | brain, testes, eyes, and livers | Rat cardiac fibroblasts [173] | variety of tissues, including kidney and colon epithelial cells, and cardiovascular and CNS nonepithelial cells |
| **Tissues (human)** | Kidney, brain (choroid plexus), lung # [174] | Liver, brain, kidney # [174] | Kidney, lung, heart, liver, and brain | Vasoconstriction; adrenal glands to release aldosterone; pituitary gland to release antidiuretic hormone (ADH, or vasopressin). | Kidney, heart, brain | the adrenal gland, kidney, lung, and heart, as brain [171] | brain, testes, eyes, and livers [175] | Heart, lung, kidney, brain, and liver | Heart, kidney, brain, and vascular wall [176] |
| **Systemic Effects** | regulator of blood pressure, systemic vascular resistance | kidney developmental abnormalities, atherosclerosis, and obesity [177] | No known systemic activity [170]. | Cardiovascular system, kidneys, blood vessels, adrenal glands, and brain. Adrenal Pituitary gland CNS | Vasoconstriction, increase blood pressure, anti-natriuresis, hypertrophy, cell proliferation, aldosterone secretion and vasopressin release [178] | Attenuates (a) diabetic cardiomyopathy [179] and (b) doxorubicin-induced cardiotoxicity [180] | Cardiovascular system, kidneys, blood vessels, adrenal glands, and brain. Adrenal Pituitary gland CNS [181] | antihypertensive and protective against cardiovascular remodeling [182, 183] | Cardiovascular system, kidneys, blood vessels, adrenal glands, and brain. Adrenal Pituitary gland CNS [184] |

***Cell**: This term is defined as either (i) selected cell-based experimental systems reported in the literature to investigate the biology of the molecules of interests or (ii) cells known to be positive for expression of the molecules of interests.

**#Data source**: The Human Protein Atlas (www.proteinatlas.org).
